# Functional Outcomes in Anterior Cruciate Ligament (ACL) Reconstruction: A Nine-Month Follow-up Study Using Lysholm Score in a Rural Tertiary Care Center in India

**DOI:** 10.7759/cureus.53480

**Published:** 2024-02-03

**Authors:** Sanjay Soni, Vinit Brahmbhatt, Mohit Tolani, Hemant Soni, Sohilkhan R Pathan, Manan Shroff, Kruti B Sharma

**Affiliations:** 1 Department of Orthopedics, Pramukhswami Medical College, Bhaikaka University, Anand, IND; 2 Clinical Research Services, Bhanubhai and Madhuben Patel Cardiac Center, Shree Krishna Hospital, Anand, IND

**Keywords:** neuromuscular rehabilitation, lysholm score, knee function, arthroscopic surgery, anterior cruciate ligament (acl) reconstruction

## Abstract

Introduction

The knee joint, an extraordinary feat of biomechanics, is prone to injuries, with the anterior cruciate ligament (ACL) often being a common victim. The intricate coordination of joint movements relies heavily on the ACL's screw-home mechanism, a crucial element for synchronizing knee movement with neighboring joints. Despite its indispensable role, the ACL is susceptible to injury, necessitating surgical intervention. While many patients experience positive outcomes following ACL reconstruction surgeries, a significant proportion face the challenge of procedure failure. The key to success lies in the healing process within the tibial and femoral bone tunnels. The post-ACL reconstruction phase introduces its own set of challenges, particularly in the context of returning to sports (RTS), underscoring the importance of reinstating neuromuscular and motor function. The trajectory of rehabilitation is influenced by factors such as graft healing, patient age, gender, pain levels, and concurrent injuries.

Materials and methods

This prospective observational study spanned 2.5 years, enrolling 71 patients with diagnosed ACL injuries. Arthroscopic reconstruction utilized hamstring autografts and peroneus longus autografts. A nine-month post-surgery follow-up employed the Lysholm scoring system for comprehensive evaluations.

Results

Over 2.5 years, 87.3% of male and 12.6% of female participants underwent arthroscopic reconstruction. Lysholm scores revealed 28.1% excellent, 45.0% good, and 26.7% fair outcomes, with no participants in the unsatisfactory range. Lysholm scores demonstrated positive outcomes, indicating the efficacy of arthroscopic reconstruction in enhancing knee function. Findings align with existing literature, emphasizing positive results from ACL reconstruction techniques and specific implants. Comparisons with related studies highlight challenges in standardized return-to-sport guidelines and underscore the need for outcome measure standardization.

Conclusion

The study contributes nuanced insights into ACL reconstruction outcomes, emphasizing positive functional recovery trends at the nine-month follow-up. Lysholm scores indicate favorable outcomes, supporting the procedure's effectiveness. Consideration of specific implants adds practical value. Despite limitations, this study enriches ACL reconstruction research, promoting advancements in patient care and outcomes. Ongoing research with extended follow-ups and larger cohorts will enhance understanding and refine ACL reconstruction strategies.

## Introduction

The knee joint, intricately designed biomechanically, is susceptible to injuries, particularly the anterior cruciate ligament (ACL), a critical component coordinating knee joint motion [1]. Despite its importance, the ACL is prone to injuries, necessitating surgical interventions for restoration.

The occurrence of ACL damage is a significant concern, estimated to range between 0.24 and 0.34 per 1,000 individuals annually [2]. This has emphasized the importance of ACL reconstruction surgeries, with about 80%-100% of patients reporting regular to almost regular outcomes post-reconstruction [3]. However, this positive outlook is tempered by the recognition that approximately 3%-10% of ACL reconstructions face failure [4]. Success in ACL reconstruction hinges on the complex recovery process in the bone tunnels, especially the tibial and femoral tunnels [5].

Beyond immediate consequences, the aftermath of ACL reconstruction introduces challenges, with the risk of subsequent injuries, including re-injury and graft failure, escalating at least tenfold compared to the risk after the initial ACL rupture [6-8]. This heightened vulnerability becomes particularly apparent during the sensitive return-to-sports (RTS) phase, aiming to reintegrate athletes without subjecting them to an overly high risk of subsequent rupture.

Critical to the RTS launch is the recovery of neuromuscular and motor function, extending beyond graft recuperation and psychosocial readiness [9-11]. Functional assessments, incorporating clinical tests and hop/leap tests, become paramount. Scrutinizing functional deficits or limb asymmetries, where the affected leg's performance is compared with the contralateral leg's performance, emerges as a predictive measure for a second ACL injury [9-11]. The enhancement of these functional skills contributes to deficit reduction, thereby reducing the following injury risk [12].

The trajectory of rehabilitation, RTS, and re-injury prevention is intricately tied to the biology of graft recuperation and maturation. The variability in individual development regarding organic recuperation, functional skills, and mental readiness introduces complexity into the timeline before RTS [13]. While defining precise time points for achieving goals or functional skills remains elusive, the temporal aspect, both before and after reconstruction, emerges as a pivotal factor in ACL reconstructions [14].

The realm of ACL reconstruction offers various graft types, with hamstring tendon autografts being the preferred choice, especially in Europe [15,16]. A newer contender is the graft based on quadriceps tendons, gaining traction due to similar clinical and functional outcomes, improved graft survival rates, and reduced harvest site pain compared to patellar autografts [17].

The complexity of factors influencing neuromuscular function post-ACL reconstruction extends beyond immediate surgical considerations. Age, sex/gender, pain intensity, and concomitant knee injuries weave into the narrative of assessing neuromuscular function after ACL reconstruction [18-20]. The multitude of these factors, engaged in a complex dance over time, underscores the need for individualized procedures in assessing functional skills. It accentuates the pivotal role of key contributors to these skills in managing deficit-oriented, function-based rehabilitation techniques post-ACL reconstructions [21,22].

## Materials and methods

Study design

In the prospective observational study conducted from 2021 to 2023, the primary focus centered on individuals diagnosed with ACL injuries. The main intervention involved a sophisticated procedure of arthroscopic reconstruction utilizing autografts from the hamstring and peroneus longus. The objective was to thoroughly investigate the intricacies of functional outcomes and the overall success trajectory of the surgery, carefully examined during an extensive nine-month postoperative follow-up period.

The intentional extension of the follow-up to nine months was a thoughtful methodological decision, allowing the research team a comprehensive timeframe to assess various outcomes. Parameters including knee stability, range of motion, muscle strength, and participants' proficiency in daily activities or sports involvement were likely taken into account. The study extended beyond objective metrics, integrating subjective measures like pain evaluation, participant satisfaction with the surgical intervention, and a comprehensive assessment of the impact on their quality of life. By encompassing both quantitative and qualitative aspects, the study endeavors to provide a thorough understanding of the sustained effectiveness and multifaceted implications associated with arthroscopic reconstruction for ACL injuries.

Study population

A total of 71 patients diagnosed with ACL injury were enrolled in the study. Informed consent was obtained from each participant before their inclusion in the research. The inclusion criteria comprised individuals with a confirmed diagnosis of ACL injury, necessitating surgical intervention. Patients with a history of previous knee surgeries or comorbidities affecting the study outcomes were excluded.

Arthroscopic reconstruction was performed on all participants using hamstring autografts and peroneus longus autografts. The surgical procedure followed established protocols and guidelines. Careful attention was given to the preparation of the graft, tunnel creation in the tibial and femoral regions, and secure fixation of the graft in the anatomical position.

A standardized follow-up protocol was implemented over a nine-month post-surgery period. The Lysholm scoring system, a validated tool for assessing knee function and outcomes, was employed for the prospective evaluation of participants. The Lysholm scoring system encompasses various parameters, offering a comprehensive assessment of knee functionality and patient-reported outcomes.

Outcome measures

The meticulous evaluation of functional outcomes in individuals undergoing arthroscopic reconstruction for ACL injury, utilizing hamstring and peroneus longus autografts, was a methodical and comprehensive process. This detailed examination, conducted over a nine-month post-surgery period, involved a systematic assessment employing a diverse set of validated parameters to scrutinize key outcome measures.

Of particular significance in this thorough evaluation was the deliberate focus on the Lysholm score, a well-established and extensively validated knee scoring system [23]. This scoring mechanism assumed a pivotal role, serving as a critical instrument in providing an intricate and in-depth assessment of the overall knee function and symptomatology experienced by individuals undergoing ACL reconstruction. As a comprehensive framework, the Lysholm score took into careful consideration a spectrum of influential factors that significantly impact knee function, ranging from pain and instability to limitations in daily activities. Pain, recognized as a fundamental aspect of the postoperative recovery process, underwent a meticulous analysis, offering nuanced insights into the subjective experiences of discomfort reported by patients. The Lysholm score also encompassed critical factors such as instability, essential for comprehending the restoration of joint stability post-surgery, and limitations in daily activities, providing a nuanced perspective on the practical repercussions of the procedure on patients' daily lives. Through the integration of these diverse elements, the Lysholm score played an instrumental role in contributing to a holistic comprehension of the multifaceted nature of knee function post-arthroscopic reconstruction. This detailed and thorough evaluation, encompassing not only pain and stability but also restrictions in daily activities, injected a layer of precision into the assessment process. The insights derived from this study, enriched by the meticulous examination facilitated by the Lysholm score, offered a nuanced and detailed portrayal of the overall efficacy of the arthroscopic reconstruction surgical intervention over the extended nine-month follow-up period.

## Results

**Figure 1:**
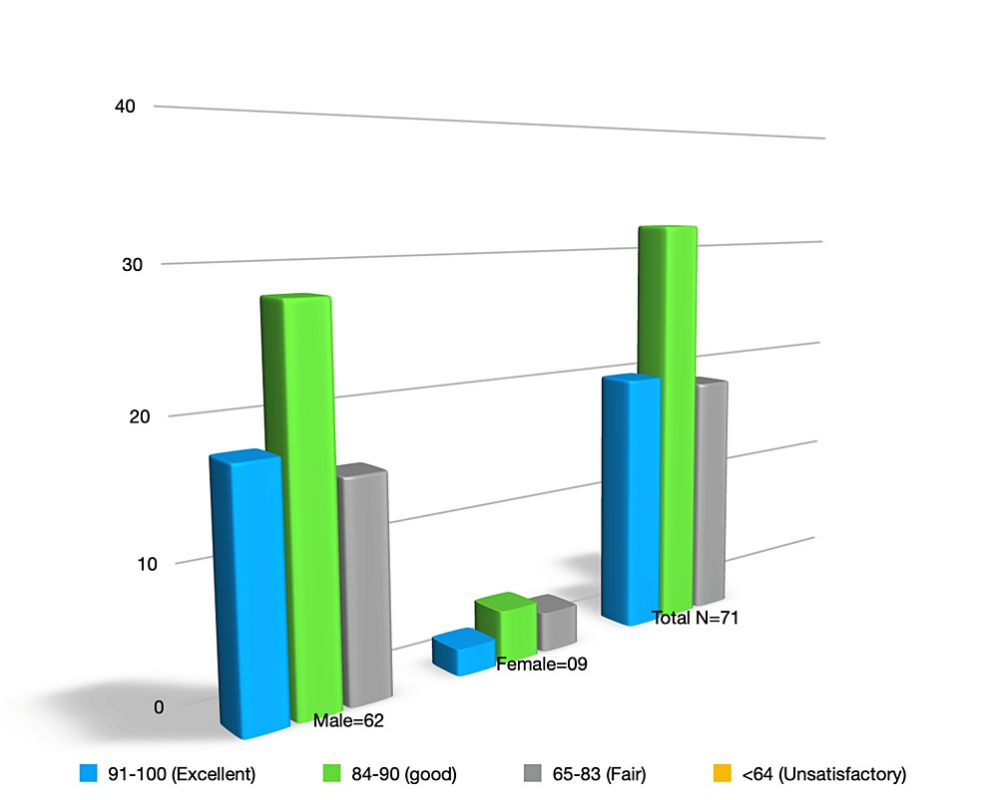
Lysholm scale categories

A total of patients, spanning the years 2021 to 2023, underwent arthroscopic reconstruction for ACL injury, with 87.3% being male and 12.6% female. This gender distribution reflects a typical prevalence of ACL injuries observed in the literature, highlighting the predominance of males in ACL reconstruction procedures. Over the 2.5-year period, participants were subjected to a rigorous follow-up regimen, with assessments conducted at nine-month intervals to evaluate their functional outcomes using the Lysholm scoring system. The results were graphically represented using a bar graph, illustrating Lysholm Scale categories: 91-100 (Excellent), 84-90 (Good), 65-83 (Fair), and <64 (Unsatisfactory) (Figure 1).

Lysholm scale distribution

The evaluation of functional outcomes, assessed through the Lysholm scale, revealed a diverse distribution of scores among the participants. A notable 28.1% of patients achieved scores within the excellent range, showcasing a substantial proportion of individuals experiencing a high level of functional recovery post-ACL reconstruction. The majority of patients, comprising 45.0%, fell within the good range on the Lysholm scale. This suggests a prevalent trend toward favorable functional outcomes in a substantial portion of the study cohort. Approximately 26.7% of patients demonstrated fair functional outcomes, indicating a moderate level of recovery following ACL reconstruction. Encouragingly, no patients scored within the unsatisfactory range, suggesting that the procedure yielded generally positive results across the study cohort. This suggests an overall positive trend in postoperative outcomes, with none of the participants experiencing unsatisfactory functional results (Table 1).

**Table 1:** Lysholm scores, ranging from 0 to 100, categorize functional outcomes into excellent, good, fair, and unsatisfactory. Higher scores indicate better postoperative recovery, encompassing various aspects of knee function and stability.

Lysholm Scale	Male=62	Female=09	Total N=71
91-100 (Excellent)	18 (25.3%)	2 (2.8%)	20 (28.1%)
84-90 (good)	28 (39.4%)	4 (5.6%)	32 (45.0%)
65-83 (Fair)	16 (22.5 %)	3 (4.2 %)	19 (26.7%)
<64 (Unsatisfactory)	0	0	0

The Lysholm scores attained by the study participants collectively underscore the clinical significance of arthroscopic reconstruction in ACL injury management. The prevalence of excellent and good outcomes indicates that a significant proportion of patients experienced substantial functional recovery, contributing to improved quality of life and potentially facilitating a return to pre-injury activity levels.

The absence of patients in the unsatisfactory category suggests a positive trajectory in ACL reconstruction outcomes. Further research, employing larger sample sizes and perhaps incorporating longitudinal assessments, could provide deeper insights into the long-term implications of these functional outcomes. Understanding the factors contributing to variations in Lysholm scores may inform personalized rehabilitation strategies and contribute to advancements in ACL injury management.

In summary, the Lysholm scores present a nuanced perspective on the functional outcomes following arthroscopic reconstruction for ACL injury. The distribution across different score ranges, combined with a gender-specific analysis, enriches our understanding of the complexities involved in postoperative recovery.

## Discussion

The examination of Lysholm scores in our study sheds light on the diverse spectrum of functional outcomes following arthroscopic reconstruction for ACL injury. The absence of patients in the unsatisfactory range signifies an encouraging trend in postoperative outcomes. The Lysholm scores demonstrate that the majority of patients experienced satisfactory to excellent functional recovery, reflecting the success of arthroscopic reconstruction in enhancing overall knee function. The distribution of Lysholm scores, particularly the significant proportion in the excellent and good categories, has immediate implications for clinical practice. These findings underscore the effectiveness of arthroscopic reconstruction in promoting positive functional outcomes and restoring the overall quality of life for a substantial number of patients. Orthopedic practitioners can leverage these insights to refine postoperative care strategies and optimize patient recovery. The Lysholm scores obtained in our study contribute to the growing body of evidence supporting the efficacy of arthroscopic reconstruction as a viable intervention for ACL injuries. The absence of patients in the unsatisfactory range reinforces the procedure's positive impact on knee function and stability.

Our study and the research by Sharma et al. [24] underscore the success of ACL reconstruction in yielding positive functional outcomes. Both investigations demonstrated significant improvements in Tegner activity levels and pain scores post-surgery. Patient-reported outcomes (PROs), including International Knee Documentation Committee (IKDC) and Lysholm scores, consistently fell within the category of good knee status and function, indicating satisfactory postoperative results. Notably, both studies attribute these positive outcomes to the use of specific implants, such as titanium adjustable loop buttons and Poly-L-co-DL-Lactic Acid-Beta Tricalcium Phosphate (PLDLA-bTCP) interference screws. While acknowledging the inherent variations in study designs and patient populations, these parallel findings collectively emphasize the efficacy of ACL reconstruction techniques and highlight the potential success of these implant choices in achieving optimal functional recovery.

In contrast to the study by Harris et al. [25], our research focuses on specific functional outcomes, namely Lysholm scores, following ACL reconstruction, while Harris et al. investigate the return-to-sport guidelines in Level I randomized controlled trials. Both studies underscore challenges in establishing standardized criteria for a safe return to sport after ACL reconstruction. Harris et al. highlight the infrequent use of objective guidelines and the variability in reported permission to resume sports activities. In our study, while not directly addressing return-to-sport guidelines, the positive functional outcomes, as indicated by Lysholm scores, contribute valuable insights into the overall success of ACL reconstruction. Both studies emphasize the need for further research and validated guidelines to guide clinical practice and ensure a safe and effective return to sport post-ACL reconstruction.

Compared to the systematic review by Berk et al. [26], our study contributes to the understanding of PROs after ACL reconstruction by specifically focusing on Lysholm scores. Berk et al. highlight the marked heterogeneity and inconsistency in the utilization of validated PROs across 510 studies, emphasizing the need for greater standardization in outcome reporting. While our study aligns with the general trend of assessing outcomes post-ACL reconstruction, the specificity of our focus on Lysholm scores provides valuable insights into a particular facet of functional recovery. The review's emphasis on the limited reporting of return-to-sport rates resonates with the broader challenge in the literature, underscoring the necessity for standardized outcome measures to facilitate meaningful comparisons and a comprehensive understanding of ACL reconstruction outcomes.

Our study on ACL reconstruction outcomes at the nine-month follow-up emphasizes Lysholm scores, revealing positive results and confirming the procedure's effectiveness in promoting favorable recovery. The use of specific implants, including titanium adjustable loop buttons and PLDLA-bTCP interference screws, adds practical insights. This research contributes valuable information for clinicians, showcasing the success of ACL reconstruction in improving clinical and functional status, considering factors like pain, swelling, and specific activities.

Despite the positive outcomes observed, it is crucial to acknowledge certain limitations in our study. The relatively modest sample size may impact the generalizability of findings, and the absence of a control group limits our ability to make direct comparisons. Additionally, the nine-month follow-up duration provides a snapshot of mid-term outcomes, and a more extended follow-up period would offer insights into the long-term durability of the surgical intervention.

## Conclusions

In conclusion, our study delving into ACL reconstruction outcomes at the nine-month follow-up offers a nuanced perspective on functional recovery. The focus on Lysholm scores as a primary measure indicates positive outcomes, affirming the efficacy of ACL reconstruction in enhancing knee function and stability. The utilization of specific implants, namely titanium adjustable loop buttons and Poly-L-co-DL-Lactic Acid-Beta Tricalcium Phosphate (PLDLA-bTCP) interference screws, underscores their potential as viable options for successful surgical interventions. The observed improvements in clinical parameters, including pain, swelling, and functional activities like climbing stairs and squatting, provide clinicians with valuable insights into the holistic recovery of patients undergoing ACL reconstruction. While the study's positive findings are promising, it is essential to acknowledge certain limitations, including the modest sample size and the absence of a control group. Additionally, the mid-term follow-up duration at 9 months limits our understanding of the long-term durability of the surgical intervention. Nevertheless, our findings contribute to the evolving landscape of ACL reconstruction research, emphasizing the significance of patient-reported functional outcomes. The study adds to the growing body of evidence supporting the positive impact of ACL reconstruction on patients' overall well-being. As we move forward, continued research efforts, with extended follow-up periods and larger cohorts, will provide a more comprehensive understanding of the sustained benefits and potential areas for refinement in ACL reconstruction techniques and rehabilitation strategies. Overall, our study contributes to the ongoing dialogue in orthopedic research, fostering advancements that ultimately aim to enhance the quality of care and outcomes for individuals undergoing ACL reconstruction.
